# Evaluation of synthesized biosurfactants as promising corrosion inhibitors and alternative antibacterial and antidermatophytes agents

**DOI:** 10.1038/s41598-023-29715-5

**Published:** 2023-02-14

**Authors:** Ahmed Fawzy, Areej Al Bahir, Nada Alqarni, Arafat Toghan, Manal Khider, Ibrahim M. Ibrahim, Hussein Hasan Abulreesh, Khaled Elbanna

**Affiliations:** 1 Department of Chemistry, Faculty of Applied Science, Umm Al-Qura University, Makkah, 21955 Saudi Arabia; 2 Chemistry Department, Faculty of Science, Assiut University, Assiut, 71516 Egypt; 3 Chemistry Department, Faculty of Science, King Khalid University, Abha, 64734 Saudi Arabia; 4 Chemistry Department, College of Science and Arts in Balgarn, University of Bisha, Bisha, 61922 Saudi Arabia; 5 Chemistry Department, College of Science, Imam Mohammad Ibn Saud Islamic University (IMSIU), Riyadh, 11623 Saudi Arabia; 6 Chemistry Department, Faculty of Science, South Valley University, Qena, 83523 Egypt; 7 Department of Dairy Science, Faculty of Agriculture, Fayoum University, Fayoum, 63514 Egypt; 8 Department of Agricultural Microbiology, Faculty of Agriculture, Fayoum University, Fayoum, 63514 Egypt; 9 Department of Biology, Faculty of Applied Science, Umm Al-Qura University, Makkah, Saudi Arabia; 10 Research Laboratories Unit, Faculty of Applied Science, Umm Al-Qura University, Makkah, Saudi Arabia

**Keywords:** Biological techniques, Chemical biology, Microbiology, Chemistry

## Abstract

This study investigated different amino acid-based surfactants (**AASs**), also known as biosurfactants, including sodium N-dodecyl asparagine (**AS**), sodium N-dodecyl tryptophan (**TS**), and sodium N-dodecyl histidine (**HS**) for their potential anticorrosion, antibacterial, and antidermatophyte properties. The chemical and electrochemical techniques were employed to examine the copper corrosion inhibition efficacy in H_2_SO_4_ (1.0 M) solution at 298 K. The results indicated their promising corrosion inhibition efficiencies (% IEs), which varied with the biosurfactant structures and concentrations, and the concentrations of corrosive medium. Higher % IEs values were attributed to the surfactant adsorption on the copper surface and the production of a protective film. The adsorption was in agreement with Langmuir adsorption isotherm. The kinetics and mechanisms of copper corrosion and its inhibition by the examined **AASs** were illuminated. The surfactants behaved as mixed-kind inhibitors with minor anodic priority. The values of % IEs gained from weight loss technique at a 500 ppm of the tested surfactants were set to be 81, 83 and 88 for **AS, HS** and **TS**, respectively. The values of % IEs acquired from all the applied techniques were almost consistent which were increased in the order: **TS > HS ≥ AS**, establishing the validity of this study. These surfactants also exhibited strong broad-spectrum activities against pathogenic Gram-negative and Gram-positive bacteria and dermatophytes. **HS** exhibited the highest antimicrobial activity followed by **TS**, and **AS**. The sensitivity of pathogenic bacteria varied against tested **AASs**. *Shigella dysenteriae* and *Trichophyton mantigrophytes* were found to be the most sensitive pathogens. **HS** exhibited the highest antibacterial activity against *Shigella dysenteriae, Bacillus cereus, E. coli*, *K. pneumoniae,* and* S. aureus* through the formation of clear zones of 70, 50, 40, 39, and 35 mm diameters, respectively*.* **AASs** also exhibited strong antifungal activity against all the tested dermatophyte molds and fungi. **HS** caused the inhibition zones of 62, 57, 56, 48, and 36 mm diameters against *Trichophyton mantigrophytes*, *Trichophyton rubrum, Candida albicans, Trichosporon cataneum,* and *Cryptococcus neoformans*, respectively. **AASs** minimal lethal concentrations ranged between 16 to 128 µg/ml. **HS** presented the lowest value (16 µg/ml) against tested pathogens followed by **TS** (64 µg/ml), and **AS** (128 µg/ml). Therefore, **AASs**, especially **HS,** could serve as an effective alternative antimicrobial agent against food-borne pathogenic bacteria and skin infections-associated dermatophyte fungi.

## Introduction

Metallic corrosion, a natural process, is the degradation of metal structures, strength, and appearance leading to huge losses to the global economy^1–6^. Corrosion inhibitors are employed for protection of metallic surfaces^7–9^. They are special compounds containing certain functional groups, aromatic and/or heterocyclic rings, plane conjugated structures and heteroatoms. These features support their adsorption on the metallic surfaces that also determine the efficiencies of the inhibitors^10–12^. Due to the interface and surface impacting properties, surfactants are extensively employed in various vital industrial applications^13–17^. The lower critical micelle concentration values of surfactants facilitate their migration and adsorption to the surfaces and inhibit metallic surface corrosion^18,19^. For this reason, numerous surfactants have been employed as corrosion inhibitors for metallic materials protection against corrosion^20–24^. Natural amino acids and fatty acids or their derivatives from the oleochemical source are condensed to prepare biodegradable and biocompatible amino acids-based surfactants (**AASs**)^14–16^. Lower toxicity, emulsifying properties, and better surface activity of **AASs** enable their food applications^25–27^. The better antifungal, antimicrobial, surface-activity, and environment-friendly properties of **AASs** as compared to traditional surfactants urge the researchers to look for novel surfactants^14–16^. A few **AASs** have been previously investigated for the inhibition of metallic surfaces such as for carbon steel corrosion in neutral and alkaline aqueous media^14^, mild steel corrosion in HCl^15^, in neutral solutions^16^, and Sabic iron corrosion in different media^17^.

The substantial rise in drug-resistant microorganisms in response to unjudicial antibiotics usage demands the development of novel antimicrobial agents with better stability and efficiency against pathogens. Antibiotic resistance results in higher medication costs, mortality rates, and prolonged hospital stay. Therefore, it has emerged as a major concern for global health and food security. The reduced efficacy of antibiotics has complicated the treatments of various diseases such as tuberculosis, foodborne diseases, gonorrhoea, pneumonia, and blood poisoning^28–32^. Rapid biodegradability, low toxicity, and excellent surface-active properties of synthesized amino acid-based surfactants highlight their promising role as effective alternatives to conventional antimicrobial agents. They are also widely used in food, cosmetics, pharmaceutical, liposome formation, and drug delivery applications as emulsifiers, softeners, wetting agents, detergents, and transfection vectors. The utility of **AASs** in healthcare interventions such as transplants, surgery, and cancer treatments has also been examined^33,34^. Structurally, **AASs** are analogues of native lipopeptides, which are cationic amphiphiles consisting of one or two amino acids linked to a hydrophobic moiety. Therefore, they are less susceptible to developing resistance and possess a similar mechanism against microorganisms^34–36^. Antibiotic resistance has been reported in many bacterial pathogens including *Staphylococcus aureus, Neisseria gonorrhoeae, Mycobacterium tuberculosis, Enterococcus faecium, Klebsiella pneumoniae, Shigella*, *Acinetobacter baumannii, Salmonella*, and *Pseudomonas aeruginosa*^29,32–34,37^. The discovery and development of new antimicrobial agents have become a prerequisite to countering the rising drug resistance in fungi and bacteria.

Based on the above-mentioned arguments, this study evaluated the corrosion inhibition efficiencies of three synthesized AASs (structures illustrated below), for the first time, for copper, which is a strategic metal in various industrial applications. The aggressive acidic media (H_2_SO_4_ solution) was employed during this study that is widely used for copper descaling, pickling, and cleaning. Different techniques including weight loss (WL), potentiodynamic polarization (PDP) and electrochemical impedance spectroscopy (EIS) were applied during the study. The study further elaborated on the kinetics and mechanisms of copper corrosion and its inhibition by **AASs**. Antibacterial and antifungal activities of the tested surfactants were also analyzed.
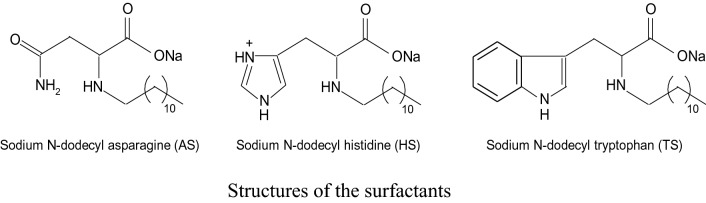


## Experimental section

### Materials and corrosion measurements techniques

Analytical grade chemicals were used in the study and the reagents’ solutions were prepared in dd H_2_O·H_2_SO_4_ (1.0 M) solution was selected to serve as the principal corrosive medium. The surfactants were prepared according to Fawzy et al.^14^ and their used concentrations ranged between 100 and 500 ppm (mg/l). Copper specimens (Merck) containing other metals such as Fe (0.030%), Pb (0.021%), Ni (0.011%), and Si (0.005%) were used for the corrosion tests. The experiments were conducted in stagnant and aerated media in triplicate to test the reproducibility, and for examine the latter the values of standard deviation (SD) of the acquired outcomes were also calculated.

Weight loss (WL), potentiodynamic polarization (PDP) and electrochemical impedance spectroscopy (EIS) techniques were followed during the study. The copper specimen surface was polished before experiments with various grades of emery papers (up to 1500), degreased with acetone, washed with dd H_2_O, and air-dried. Copper sheets with dimensions 3.6 × 1.4 × 0.2 cm^2^ were utilized in the WL method whereas a cylinder-shaped rod (1.0 cm^2^) enclosed in Araldite was used for PDP and EIS techniques.

During WL analysis, the prepared copper sheets were inserted in 100 ml of the corrosive solutions (1.0 M H_2_SO_4_) and in the presence of different concentrations of **AASs** for a fixed time intervals each 4 h. Then, the copper sheets were degreased, washed, air-dried and weighted to assess the average weight loss (mg/cm^2^). For PDP and EIS measurements, the copper electrode was prepared as reported earlier^38–40^ and was immersed in a cell containing the examined medium at open circuit potential (OCP). Thermostated PGSTAT30 potentiostat/galvanostat was used to record the electrochemical measurements. The cell contained a working electrode of the copper specimen, a counter electrode of the platinum sheet, and a reference electrode of calomel. A frequency range of 100 kHz to 0.1 Hz and amplitude of 5.0 mV (peak to peak) was set in EIS studies, which utilized AC signals at OCP.

### Media and strains used in this study

To determine the antimicrobial activity of AAS, *S. aureus* (ATCC25923) and *Bacillus cereus* (ATCC11778) were used as Gram-positive whereas *E. coli* (ATCC25922), *K. pneumoniae* (ATCC No. 700603), and *Shigella dysenteriae* (DSM103303) were used as Gram-negative bacterial indicators. AAS were also evaluated against skin infections-associated dermatophyte fungi including *Trichophyton mantigrophytes* (ATCC No. 18748), *Trichophyton rubrum* (ATCC No. 28188)*, Trichosporon cataneum* ATCC No. 28592, *Cryptococcus neoformans* (ATCC No. 208821), and *C. albicans* (ATCC No.90028). All bacterial strains were obtained from the Bacterial Culture Collection of Agricultural Microbiology Department, Faculty of Agriculture, Fayoum University, Egypt. All the fungal isolates were obtained from Fungi Center, Assiut University, Egypt. Bacterial stock cultures were maintained at 4 °C on Mueller Hinton agar plates whereas moulds and fungi were sub-cultured on potato dextrose agar plates and maintained at 4 °C.

### Assessment of antimicrobial activity

Agar well diffusion method was adopted to assess the antimicrobial potential of tested surfactants^41,42^. Briefly, Mueller Hinton agar medium and potato dextrose agar were prepared, autoclaved at 121 °C, cooled at 50 °C, poured into sterilized Petri dishes, and solidified at room temperature. Subsequently, Mueller Hinton agar plates were swabbed with fresh bacterial cultures whereas fresh fungal cultures were swabbed onto PDA plates. A sterilized cork borer was used to create the wells (9 mm) in the center of agar plate. **AASs** stock solutions (1 mg/ml) were prepared and 100 µg of each AAS was placed inside the wells. Plates containing bacterial pathogens, *Candida albicans* yeast, and *Cryptococcus* sp. were incubated at 30 °C for 24–48 h, respectively. The incubation of remaining fungal pathogens was carried out for 48–72 h at 28 °C. The diameter of inhibition zones (mm) around each well was measured to assess the **AASs** antimicrobial activity. Fluconazole, Nystatin (Mycosat), and Ciclopirox (Batrafen) (100 μg/ml) were used as antifungal standards whereas water treatment served as control. Bacterial inhibition (mm) zones were measured by following the agar disk diffusion method and compared with antibiotic standards (used against Gram-negative and positive bacteria)^43^.

### Estimation of minimum lethal concentrations (MLC)

The dilution method was followed to determine the Minimum lethal concentrations (MLCs) of **AASs**^44^. To determine the MLC of *Candida albicans* and bacteria, Two-fold serial concentrations of **AAS**s were pipetted into tubes containing potato dextrose broth media (PD) or LB (4 ml), respectively. 0.4 ml of 0.5 McFarland medium from each standard bacterial suspension containing 1 × 10^6^ cell/ml^−1^ was inoculated in each tube. To determine the MLC of fungal pathogens, two-fold serial concentrations of **AASs** were pipetted into PD broth (4 ml) containing tubes and 1 × 10^6^ spore/ml^−1^ were inoculated in each tube^41,42^. The incubation of tubes was carried out at optimum temperatures and time intervals for each microorganism. After incubation, 0.1 ml solution of each tube was sub-cultured onto Mueller Hinton agar plates or PDA plates and again incubated at their respective optimum temperatures and time intervals. The lowest **AAS** concentration producing only 0.1% viable count in comparison to the original inoculum of 1 × 10^6^ cell/ml was considered the minimal lethal concentration (MLC).

## Results and discussion

### Assessment of inhibition efficiencies

#### WL measurements

##### Impact of H_2_SO_4_ concentration on the inhibition efficiencies

**Figure 1:**
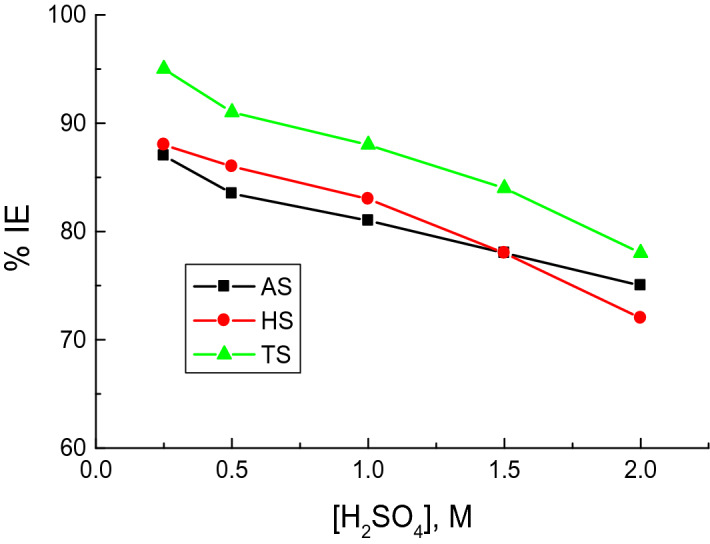
The impact of corrosive medium concentration on the values of % IEs of the examined surfactants in the copper corrosion in H_2_SO_4_ solutions at 298 K.

To study the H_2_SO_4_ impact on the corrosion inhibition efficiencies (% IEs) of the examined surfactants (**AS, HS, TS**), WL measurements were conducted using different concentrations of H_2_SO_4_ solution (0.25–2.0 M) in the presence of a fixed concentration of the surfactants (500 mg/l) at 298 K and are illustrated in Fig. 1.

Corrosion rates (CR) of copper were calculated as mils penetration per year (mpy) by applying the following Eq. (1)^45^:

1 $${\text{CR }}\left( {{\text{mpy}}} \right) = \frac{KW}{{Atd}}$$

where *K* is constant (3.45 × 10^6^), *W* represents WL (grams), *A* represents the area of the copper sheet (cm^2^), *t* represents time (hour), and *d* represents copper density.

The % IEs and the degrees of surface coverage (θ) of the **AASs** were calculated using Eq. (2)^46^:

2 $$\% {\text{ IE }} = \theta \, \times { 1}00 \, = \left[ {1 - \frac{{CR_{inh} }}{CR}} \right] \times { 1}00$$

where CR_inh_ and CR respectively represent the corrosion rates with and without inhibitor.

Figure 1 reveals that higher H_2_SO_4_ solution concentration reduced the values of % IEs, which indicates higher surfactant efficiency at lower corrosive medium concentration. These results can be attributed to the more aggressive potential of the corrosive medium at high concentrations.

##### Impact of surfactant concentrations on the corrosion rates

**Figure 2:**
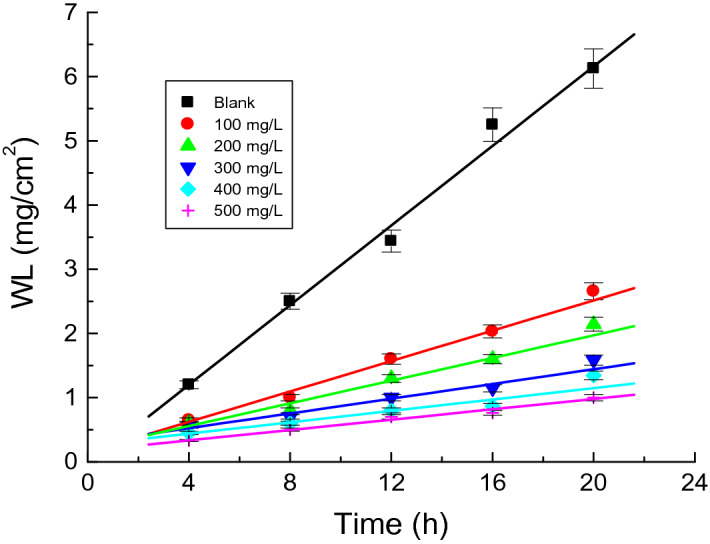
WL versus immersion time plots for the copper corrosion in 1.0 M H_2_SO_4_ solution and in the presence of the surfactant **AS** at 298 K.

**Figure 3:**
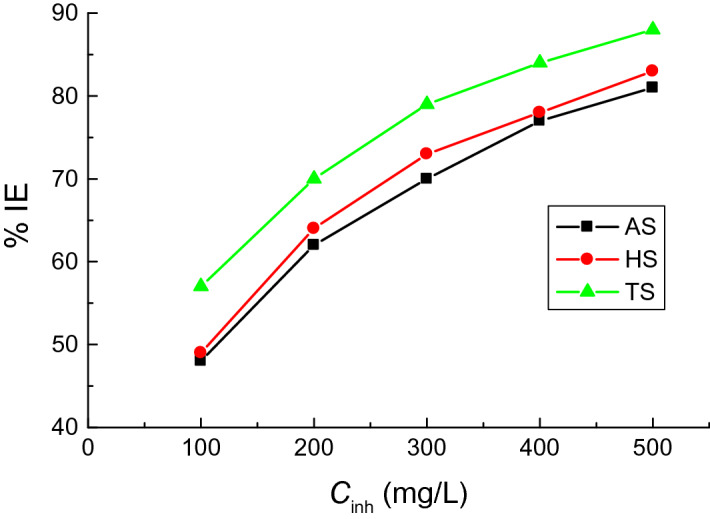
The impact of concentrations of the examined surfactants on the copper corrosion inhibition in 1.0 M H_2_SO_4_ solution at 298 K.

WL measurements for copper sheets in 1.0 M H_2_SO_4_ solution were conducted at 298 K without and with the examined **AASs** at different concentrations (100–500 mg/L). Figure 2 illustrates the WL plots of the surfactant AS versus immersion time (as a representative example). Table 1 depicts copper CRs, % IEs, and θs of tested **AASs** at 298 K. Here, the values of standard deviation (SD) of the corrosion rates were also calculated. The results revealed reduced copper CRs whereas the values of % IEs and θs of the surfactants were increased at higher concentrations. Table 1 also shows that the SD values were very low, indicating higher precision of the acquired results. These findings can be attributed to the surfactant molecule's enhanced adsorption on the copper surface, which increased at higher concentrations to reduce CRs values and increase % IEs and θs values. The tested surfactants efficiently inhibited the copper corrosion in the H_2_SO_4_ (1.0 M) solution. The values of % IEs also increased at a particular **AASs** concentration as **TS > HS ≥ AS** as illustrated in Fig. 3.

**Table 1:** Values of copper CRs in 1.0 M H_2_SO_4_ solution, % IEs and θs of the examined surfactants at 298 K.

Surf	CR (mpy)	AS			CR (mpy)	HS			CR (mpy)	TS		
Conc (mg/l)		SD	% IE	θ		SD	% IE	θ		SD	% IE	θ
0	112	5.817	–	–	112	5.817	–	–	112	5.817	–	–
100	58	2.704	48	0.482	57	3.021	49	0.491	48	1.984	57	0.571
200	43	2.541	62	0.616	40	2.243	64	0.643	34	1.802	70	0.696
300	34	1.478	70	0.696	30	1.747	73	0.732	24	1.317	79	0.786
400	26	1.402	77	0.768	25	1.521	78	0.777	18	1.134	84	0.839
500	21	1.271	81	0.812	19	1.092	83	0.830	13	0.581	88	0.884

*AS* sodium N-dodecyl asparagine, *HS* sodium N-dodecyl histidine, *TS* sodium N-dodecyl tryptophan, *CR* corrosion rates, *% IE* inhibition efficiency.

##### Adsorption consideration

**Figure 4:**
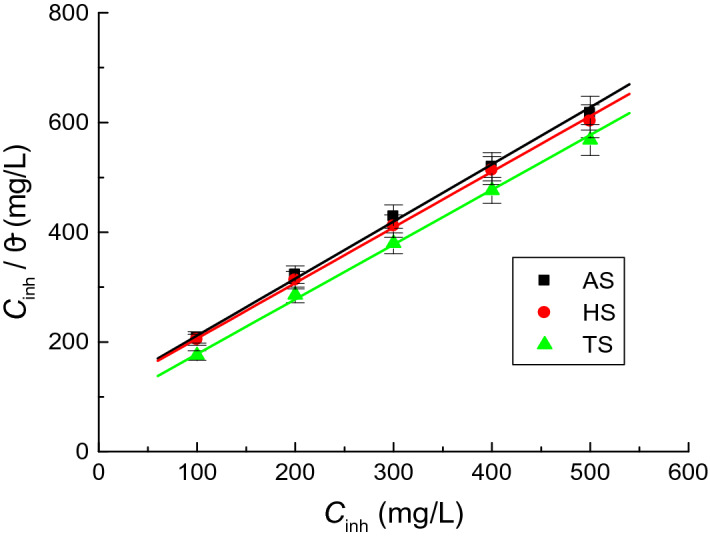
Langmuir adsorption isotherms for the adsorption of the examined surfactants on the copper surface in 1.0 M H_2_SO_4_ solution at 298 K.

**AASs** emerged as efficient inhibitors of copper corrosion in the H_2_SO_4_ (1.0 M) solution. Protective film formation and surfactant molecule adsorption on the copper surface could have contributed to their better anticorrosion activity^47–51^. The mechanism of **AASs** adsorption on the copper surface was further elaborated by subjecting θs values of different surfactant concentrations to various adsorption isotherms (Langmuir, Frumkin, Temkin, and Freundlich). Figure 4 demonstrates that results followed Langmuir isotherm and were expressed by Eq. (3)^52^,

3 $$\frac{{C_{inh}^{{}} }}{\theta } = \frac{1}{{K_{ads} }} + C_{inh}^{{}}$$

where *K*_ads_ represents the adsorption constant. Linear plot intercepts were used to calculate their values as 2.61 × 10^3^, 3.21 × 10^3^, and 4.70 × 10^3^ for **AS, HS,** and **TS**, respectively (Fig. 4).

##### Kinetics of copper corrosion and its inhibition

**Figure 5:**
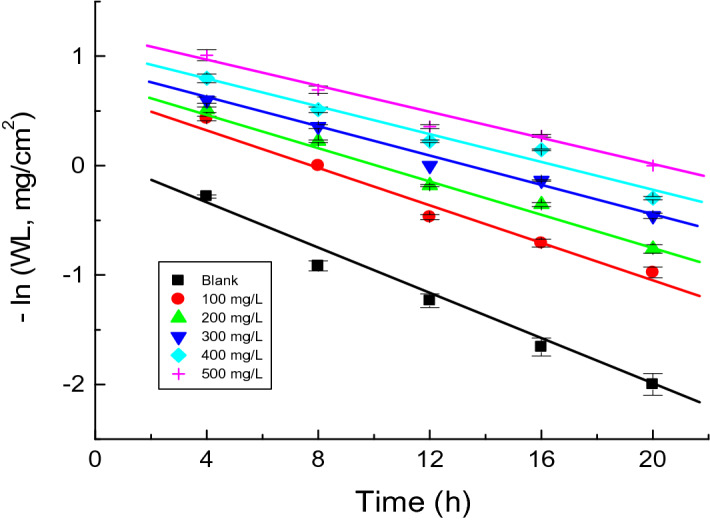
First-order rate constant plots for the copper corrosion in 1.0 M H_2_SO_4_ solution and in the presence of the surfactant **HS** at 298 K.

Kinetics of corrosion points was followed to test the corrosion inhibition features and measure the stability of chemical species found during the corrosion of various materials. The kinetics of copper corrosion was investigated in H_2_SO_4_ (1.0 M) solution along with surfactant **HS** (as an example) at 298 K. Figure 5 presents linear plots of – ln(WL) vs. time (based on the first order rate constant equation and the corrosion process). It reveals that the kinetics of copper corrosion in H_2_SO_4_ solution and its inhibition by** HS** surfactant were negative first-order processes. The gradients of such plots refer to the first-order rate constant values [*k*_1_ (in h^−1^)] (Table 2). Half-life (*t*_1/2_, h) values were calculated by following Eq. (4)^53^ (Table 2)

4 $$t_{1/2} = \frac{0.693}{{k_{1} }}$$

**Table 2:** Values of the first-order rate constant (*k*_1_) and half-life (*t*_1/2_) of copper corrosion in 1.0 M H_2_SO_4_ solution and in the presence of the surfactant **HS** at 298 K.

Conc. (mg/l)	10^3^* k*_1_, h^−1^	*t*_1/2_, h
0	101	6.93
100	88	7.87
200	71	9.90
300	65	10.66
400	62	11.18
500	59	11.74

The orders (*n*) of the copper corrosion inhibition with respect to surfactant (*C*_inh_) concentrations were computed using Eq. (5)^54^,

5 $${\text{log CR }} = {\text{ log}}k + {\text{ n log}}C_{{{\text{inh}}.}}$$

where *k* is the specific rate constant (mg/cm^2^ h).

**Figure 6:**
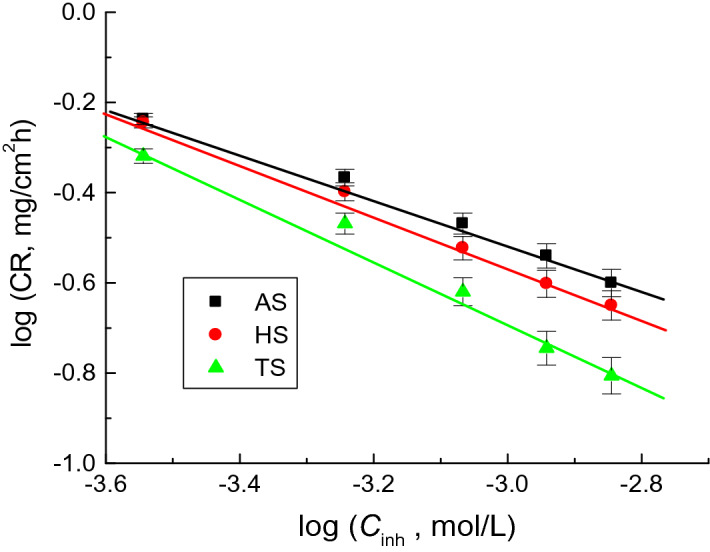
log CR vs. log *C*_inh_ for the copper corrosion inhibition by the examined surfactants in 1.0 M H_2_SO_4_ solution at 298 K.

Figure 6 exhibits linear plots of log CR versus log *C*_inh_ of tested surfactants. ***n*** values were estimated from the slopes of the plots as − 0.54, − 0.60, and − 0.71 for **AS, HS,** and **TS**, respectively. These n values of the corrosion inhibition process revealed it as a negative fractional-first order reaction with respect to the concentrations of the inhibitors. The negative *n* sign and reverse proportional CR values to the inhibitors’ concentrations refer to better % IEs of the studied surfactants^55^ (Fig. 6).

#### PDP measurements

**Figure 7:**
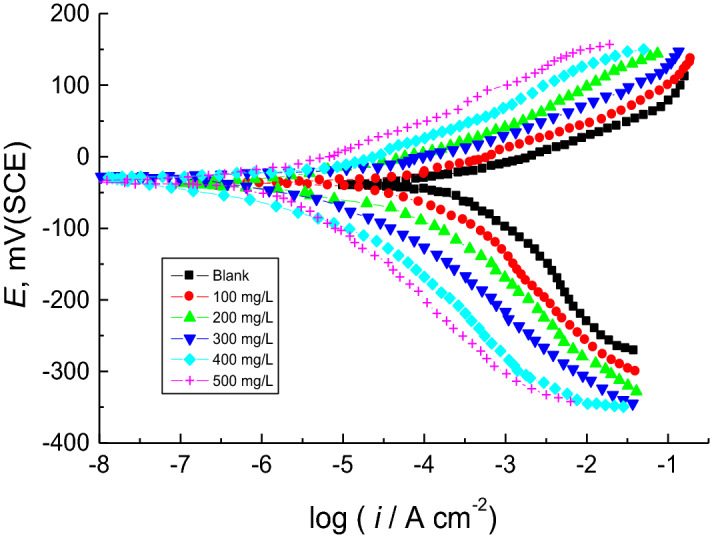
PDP curves of the copper corrosion in 1.0 M H_2_SO_4_ solution and in the presence of the surfactant **HS** at 298 K.

PDP measurements of copper corrosion were carried out in 1.0 M H_2_SO_4_ solution at 298 K in the presence and absence of different concentrations of the examined surfactants. Figure 7 only demonstrates the PDP curves (Tafel plots) of **HS** surfactant (as an example) in relevance to copper corrosion in 1.0 M H_2_SO_4_ solution. Different corrosion parameter values were obtained from Tafel plots for the examined **AASs** including corrosion potential (*E*_corr_), corrosion current density (*i*_corr_), anodic and cathodic Tafel slopes (*β*_a_, *β*_c_), and polarization resistance (*R*_p_) (Table 3). The values of % IEs of **AASs** were computed using Eq. (6) (Table 3),

6 $$\% {\text{ IE }} = \theta \, \times { 1}00 \, = \left[ {1 - \frac{{i_{corr(inh)} }}{{i_{corr} }}} \right] \times { 1}00$$

where, *i*_corr_ and *i*_corr(inh)_ represent corrosion current densities in the absence and presence of surfactants, respectively.

**Table 3:** Polarization parameters for the copper corrosion in 1.0 M H_2_SO_4_ solution and in the presence of the examined surfactants at 298 K.

Surf	Conc. (mg/l)	*− E*_corr_ (mV(SCE))	*β*_a_ (mV/dec.)	− *β*_c_ (mV/dec.)	*i*_corr_ (µA/cm^2^)	SD	*R*_p_ (ohm cm^2^)	% IE	θ
	0	44	42	95	288	26.221	44	–	–
AS	100	37	40	93	167	7.841	73	42	0.420
	200	40	39	94	107	6.014	112	63	0.628
	300	36	40	89	86	4.742	140	70	0.701
	400	34	37	87	72	4.602	157	75	0.750
	500	37	41	85	60	2.897	200	79	0.792
HS	100	42	42	83	156	7.021	78	46	0.458
	200	41	37	85	98	6.214	114	66	0.659
	300	38	39	84	84	5.127	138	71	0.708
	400	36	42	90	72	3.978	173	75	0.750
	500	41	49	92	66	2.719	211	77	0.771
TS	100	42	39	98	115	6.081	106	60	0.600
	200	39	37	88	78	4.001	145	73	0.729
	300	42	39	92	58	3.578	205	80	0.799
	400	37	41	87	46	2.397	263	84	0.840
	500	33	44	84	40	2.415	314	86	0.861

*AS* sodium N-dodecyl asparagine, *HS* sodium N-dodecyl histidine, *TS* sodium N-dodecyl tryptophan.

Figure 7 reveals that **HS** surfactant addition in the corrosive medium moved cathodic and anodic Tafel branches of the copper PDP curve in the inhibitor-free solution to lower *i*_corr_ values. The inhibition of metal dissolution in response to hindrance in cathodic and anodic reactions could be the main reason behind this phenomenon. Table 3 contains the list of corrosion parameters, which indicate that copper *E*_corr_ value recorded in the corrosive medium (blank) was slightly lowered to negative ones (towards anodic direction) when different surfactant concentrations were added. Therefore, these surfactants are considered as mixed-type inhibitors with slight anodic priority^56,57^. The *β*_a_ and *β*_c_ values were also slightly reduced in blank solution after the addition of the surfactants. It exhibits a reduction in the anodic dissolution that hinders cathodic hydrogen evolution reactions. Furthermore, copper *i*_corr_ value acquired in the corrosive solution was decreased whereas the values of both *R*_p_ and % IEs were increased at higher surfactant concentrations. Also, the values of SD of the recorded *i*_corr_ values were calculated and listed in Table 3 which illuminates lower SD values signifying higher precision of the acquired results.

#### EIS measurements

**Figure 8:**
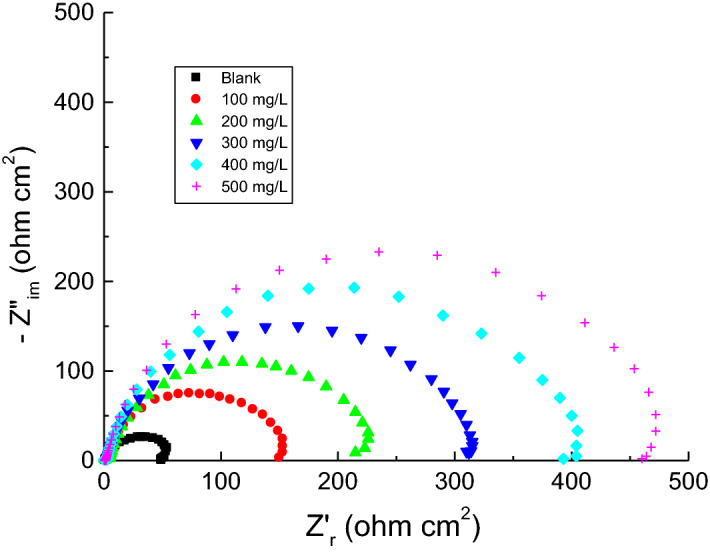
Nyquist plots representing the copper corrosion in 1.0 M H_2_SO_4_ solution and in the presence of the surfactant **TS** at 298 K.

**Figure 9:**
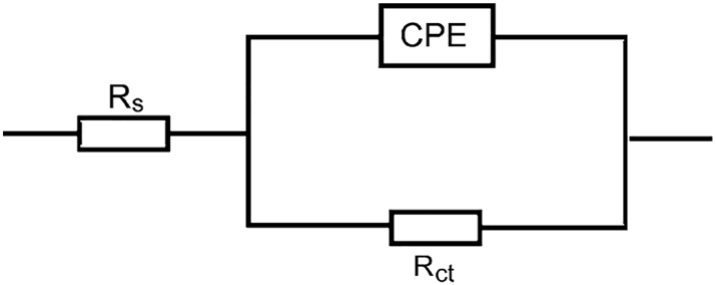
Electrochemical equivalent circuit to optimize the EIS output data for the copper corrosion in 1.0 M H_2_SO_4_ solution and in the presence of the examined surfactants.

EIS measurements for copper corrosion were performed at 298 K in 1.0 M H_2_SO_4_ solution with and without the addition of different concentrations of the tested surfactants. Figure 8 demonstrates EIS spectra (Nyquist plots) of copper corrosion in 1.0 M H_2_SO_4_ solution and in the presence of the surfactant **TS** (as an example). The spectra revealed one-time constants and single depressed capacitive loops, which indicates that double layer behavior and charge-transfer process managed the copper corrosion^58^. The size of the copper capacitive loop in the blank solution regularly increased in direct proportion to the concentrations of the examined surfactants. This behavior indicates reducing copper corrosion rates and augmenting surfactants % IEs values. The analysis of EIS spectra was carried out by comparing them with an equivalent circuit (Fig. 9). The values of constant phase element (CPE), charge transfer resistance (*R*_ct_), and solution resistance (*R*_s_) were obtained from EIS spectra and are listed in Table 4. The % IEs values were calculated by following Eq. (7)^59^ (Table 4),

7 $$\% {\text{ IE }} = \left[ {1 - \frac{{R_{ct} }}{{R_{ct(inh)} }}} \right] \times 100$$

where *R*_ct(inh)_ and *R*_ct_ represent charge transfer resistances in the absence and presence of surfactants, respectively.

**Table 4:** Impedance parameters for the copper corrosion in 1.0 M H_2_SO_4_ solution and in the presence of the examined surfactants at 298 K.

Surf	Conc. (mg/l)	*R*_s_ (ohm cm^2^)	*R*_ct_ (ohm cm^2^)	SD	CPE (µF/cm^2^)	% IE	θ
	0	1.1	57	3.701	269	–	–
AS	100	1.7	127	6.923	108	55	0.551
	200	2.1	163	7.870	97	65	0.650
	300	1.4	203	9.472	84	72	0.719
	400	0.9	238	13.214	74	76	0.761
	500	1.8	259	12.842	69	78	0.780
HS	100	1.2	124	7.084	111	54	0.540
	200	2.3	173	6.941	99	67	0.671
	300	1.7	248	11.978	75	77	0.770
	400	2.4	301	16.842	64	81	0.811
	500	1.5	356	19.047	55	84	0.840
TS	100	1.2	154	7.178	106	63	0.630
	200	1.6	228	11.951	79	75	0.750
	300	1.9	317	14.904	61	82	0.820
	400	3.8	407	22.012	48	86	0.860
	500	2.1	475	26.345	37	88	0.880

*AS* sodium N-dodecyl asparagine, *HS* sodium N-dodecyl histidine, *TS* sodium N-dodecyl tryptophan.

EIS parameter values (listed in Table 4) indicated that surfactant's addition in corrosive solution increased *R*_ct_ values and reduced CPE values. The values of SD of the acquired *R*_ct_ values were computed and listed in Table 4 which shows lower SD values signifying higher precision of the acquired results. This data confirms surfactant role as inhibitors through adsorption in copper/solution interface that subsequently shields the copper surface from corrosive solution, and then enhances the % IEs values^60–62^.

**Figure 10:**
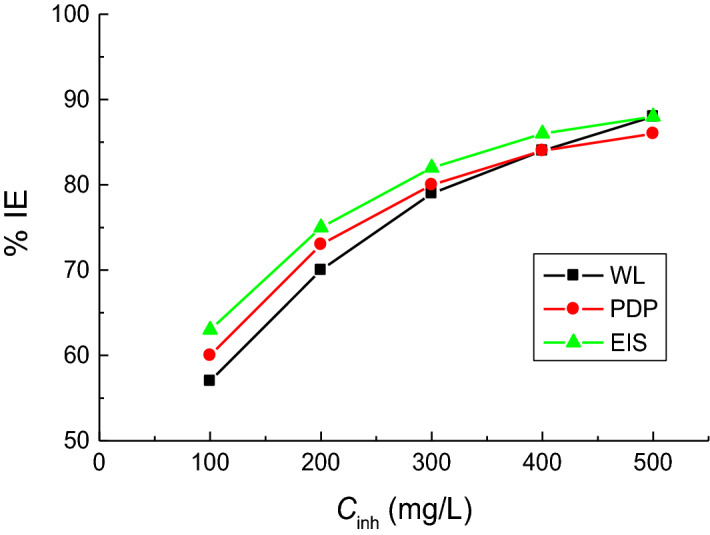
Comparison of different techniques employed for investigation of the copper corrosion inhibition by the surfactant **TS** in 1.0 M H_2_SO_4_ solution at 298 K.

Finally, the gained % IEs values of the studied surfactants obtained from the EIS technique (Table 4) were aligned to those obtained through WL and PDP techniques (Tables 1 and 3). Figure 10 depicts the values of the surfactant **TS** (as an example), which confirm the validity of employed measurements.

### Suggested mechanism for copper corrosion inhibition

Corrosion of copper in acidic media was suggested to happen through the following reactions^63^:

Anodic reaction through which copper metal is oxidized (corroded) to Cu^2+^,

8 $${\text{Cu}} = {\text{Cu}}^{{{2} + }} + {\text{ 2e}}^{ - }$$

Cathodic reaction

9 $${\text{O}}_{{2}} + {\text{ 4H}}^{ + } + {\text{ 4e}}^{ - } = {\text{2H}}_{{2}} {\text{O}}$$

The gained outcomes from the different utilized techniques; WL, PDP and EIS indicated that the examined **AASs** were discovered to be proficient inhibitors for copper corrosion in 1.0 M H_2_SO_4_ solution. The inhibition mechanism was proposed as follows. The recorded values of *E*_corr_ for copper in the tested media were positive rendering the copper surface positively charged. This permits SO_4_^2-^ ions to adsorb on the copper surface causing it becomes negatively charged. On the other hand, in acidic media the examined **AASs** which comprise polar O and N atoms are proposed to protonate to produce cations as referred by Eq. (10),

10 $${\text{AAS}} + {\text{nH}}^{ + } = \left[ {{\text{AAS }}{-}{\text{ H}}_{{\text{n}}} } \right]^{{{\text{n}} + }}$$

So, the positively charged **AASs** were adsorbed on the negative copper surface to produce solidly adsorbed film (physical adsorption). Both **AASs**’ molecules and their cations might be adsorbed on the anodic and cathodic locations, correspondingly, existed on the copper surface. Adsorption on the anodic locations could be occurred through O and N atoms leading to a delay of the copper corrosion. Adsorption on the cathodic locations results in a limitation of the O_2_ evolution^64,65^. Furthermore, complexes formation (precipitates) which could be occurred between the heteroatoms in the surfactant molecules (N & O) and unoccupied *d*-orbitals on the copper surface contribute in the adsorption process and thus in the metal corrosion inhibition^66^. So, copper corrosion inhibition could be happened by construction of two protective layers: solidly adsorbed film and/or the formed precipitate on the copper surface. The varying values of % IEs of the tested **AASs** resulting from the different adsorption capabilities of them on the copper surface was attributed to the difference in their chemical structures. The highest % IE of the surfactant **TS** could be due to the presence of indole moiety in the **TS** structure with higher adsorption properties. Also, the slight precedence of the surfactant **HS** over **AS** may be attributed to the presence of diazole ring in** HS**.

### Evaluation of antibacterial and antidermatophytes activities

**Figure 11:**
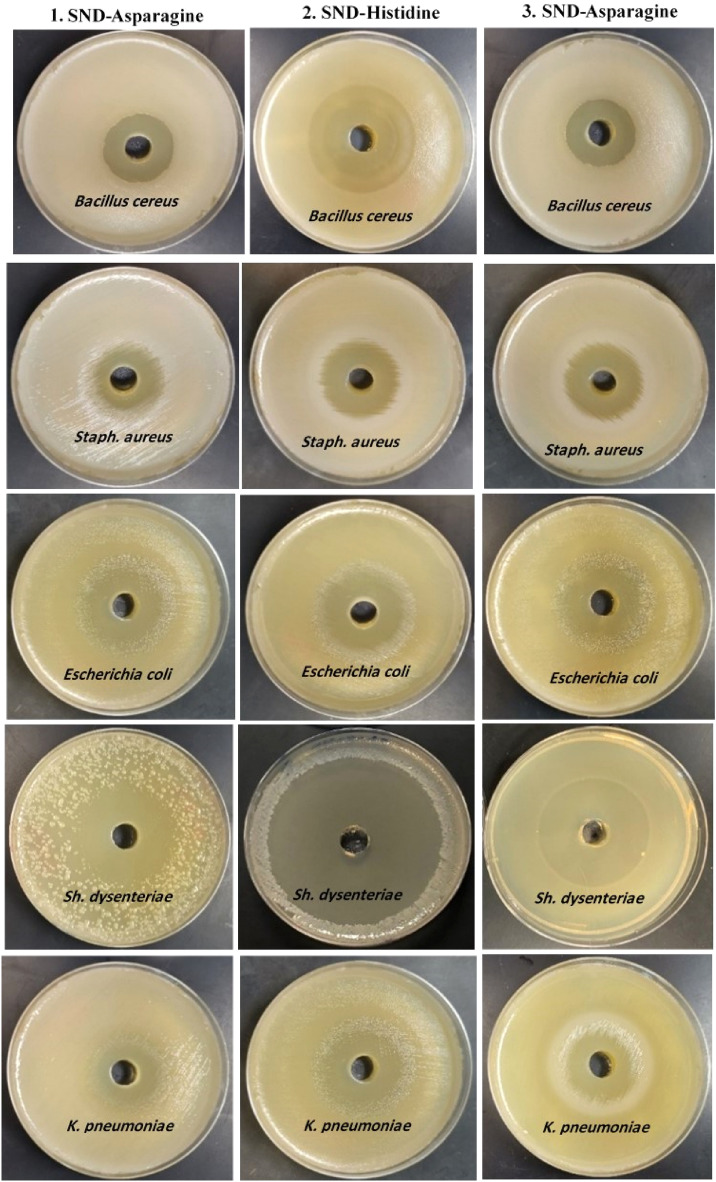
Antimicrobial activity of the surfactants in comparison to traditional antibiotics against food-borne pathogenic bacteria indicated by clear zone diameter (mm).

**Figure 12:**
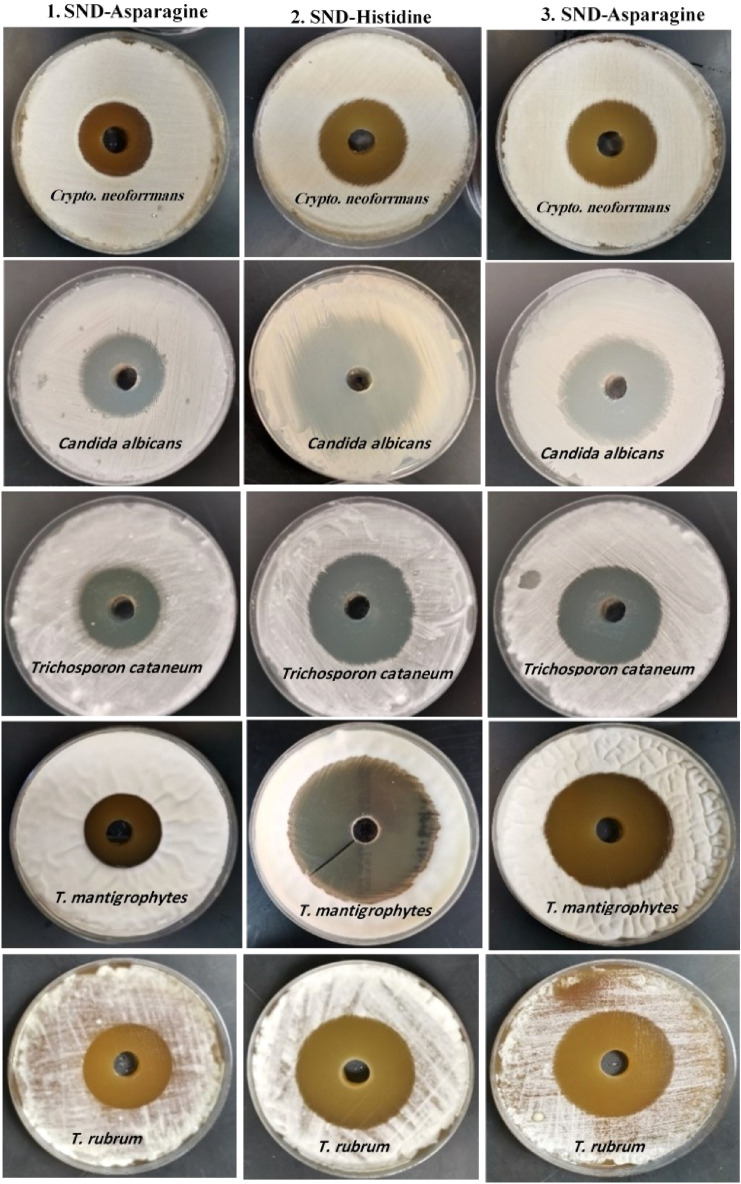
Antimicrobial activity of the surfactants in comparison to traditional antifungals against dermatophyte yeasts and fungi indicated by clear zone diameter (mm).

An alarming rise in antibiotic resistance in microbial pathogens demands the development of new, effective, stable, and highly efficient antimicrobial agents. In this scenario, the synthesized amino acid-based surfactants are gaining popularity as a better alternative to traditional antimicrobial agents and antibiotics^28,29,32,34^. All the biosurfactants tested during this study, exhibited broad-spectrum activities against Gram-negative and Gram-positive bacteria, and dermatophyte fungi through minimal lethal concentrations and clear zone inhibition (Tables 5 and 6, Figs. 11 and 12). The **HS** surfactant presented the highest antimicrobial potential followed by **TS** and **AS**. The sensitivity of various foodborne pathogenic bacteria varied against tested **AAS**. *Shigella dysenteriae* was found to be the most sensitive species followed by *Bacillus cereus, E. coli*, *K. pneumoniae,* and* S. aureus,* respectively*.* The **HS** surfactant, having a very low MLC, particularly displayed broad-spectrum activities against dermatophyte fungi and foodborne pathogenic bacteria. **HS** addition resulted in the inhibition zones of 70, 50, 40, 39, and 35 mm diameter against *Shigella dysenteriae*, *Bacillus cereus*, *E. coli*, *K. pneumoniae*, and *S. aureus* respectively whereas inhibition zones of 48, 39, 38, 34, and 33 mm diameter were noted against *Shigella dysenteriae*, *K. Pneumoniae*, *E. coli*,* S. aureus*, and* Bacillus cereus* respectively after the addition of the **TS** surfactant*.* The addition of **AS** surfactant produced the inhibition zones of 45, 36, 32, 32, and 27 mm diameter against *Shigella dysenteriae*, *E. coli*, *Bacillus cereus*, *K. pneumoniae*, and *S. aureus* respectively*.* The minimal lethal concentration of **AAS** ranged between 16 to 128 µg/ml. The lowest value (16 µg/ml) was recorded for **HS** against most of the tested pathogens followed by **TS** (64 µg/ml), and **AS** (128 µg/ml).

**Table 5:** Antimicrobial activity of the examined surfactants in comparison to traditional antibiotics against food-born pathogenic bacteria based on the clear zone diameter (CZD^a–c^, mm), and minimal lethal concentration (MLC, µg/ml).

Treatment		Clear zone diameter (mm) and minimal lethal concentration (MLC μg/ml^−1^)									
		Gram-positive bacteria				Gram-negative bacteria					
		*B. cereus*		*S. aureus*		*E. coli*		*K. pneumoniae*		*Sh. dysenteriae*	
		CZD*	MLC	CZD*	MLC	CZD*	MLC	CZD*	MLC	CZD*	MLC
Amino acids-based surfactants											
1. SND-asparagine (100 μg/ml)		32	128	27	128	36	128	32	128	45	64
2. SND-histidine (100 μg/ml)		50	16	35	16	39	16	40	16	70	16
3. SND-tryptophan (100 μg/ml)		33	64	34	64	38	64	39	128	48	32
Conventional antibiotics											
Gram-positive antibiotics											
Cephalothin 5 µg	KF	–	nd	–	nd	nd	nd	nd	nd	nd	nd
Clindamycin 2 µg	CD	12	nd	19	nd	nd	nd	nd	nd	nd	nd
Oxacillin 5 µg	OX	–	nd	–	nd	nd	nd	nd	nd	nd	nd
Cotrimoxazole 25 µg	TS	–	nd	14	nd	nd	nd	nd	nd	nd	nd
Erythromycin 5 µg	E	22	nd	–	nd	nd	nd	nd	nd	nd	nd
Gentamicin 10 µg	GM	19	nd	15	nd	nd	nd	nd	nd	nd	nd
Oxytetracycline	OT	13	nd	14	nd	nd	nd	nd	nd	nd	nd
Penicillin G 10 µg 1Unit	PG	–	nd	–	nd	nd	nd	nd	nd	nd	nd
Gram-negative antibiotics											
Amikacin 30 µg	AK	nd	nd	nd	nd	21	nd	17	nd	20	nd
Ceftazidime 30 µg	CZA	nd	nd	nd	nd	25	nd	–	nd	22	nd
Aztreonam 30 µg	ATM	nd	nd	nd	nd	30	nd	–	nd	25	nd
Piperacillin 100 µg 100 µg	PRL	nd	nd	nd	nd	15	nd	–	nd	15	nd
Imipenem 10 µg 100 µg	IMI	nd	nd	nd	nd	23	nd	20	nd	23	nd
Ciprofloxacin10 µg	CIP	nd	nd	nd	nd	29	nd	20	nd	25	nd

^a^The values are the mean of the three samples ± SD.

^b^Inhibition zone diameter was measured as the clear area in the center of agar well.

^c^*nd* not determined, –no effect.

**Table 6:** Antimicrobial activity of the examined surfactants in comparison to traditional antibiotics against molds and dermatophyte fungi based on the clear zone diameter (CZD^a–c^, mm), and minimal lethal concentration (MLC, µg/ml).

Treatment	Clear zone diameter (mm) and minimal lethal concentration (MLC μg/ml^−1^)									
	*C. albicans*		*Cryptococcus neoforrmans*		*Trichosporon cataneum*		*T. rubrum*		*T. mantigrophytes*	
	CZD*	MLC	CZD*	MLC	CZD*	MLC	CZD*	MLC	CZD*	MLC
Amino acids-based surfactants (100 μg/ml)										
1. SND-Asparagine	36	128	33	128	35	64	36	128	33	128
2. SND-Histidine	57	16	36	16	48	16	54	16	62	16
3. SND Tryptophan	45	64	36	64	40	64	43	64	35	64
Conventional antifungal (100 μg/mL)										
Fluconazole	35	nd	37	nd	38	nd	27	nd	35	nd
Nystatin	30	nd	33	nd	42	nd	28	nd	40	nd
Ciclopirox	33	nd	31	nd	38	nd	26	nd	45	nd

^a^The values are the mean of three samples ± SD.

^b^Inhibition zone was measured as the clear area in the center of agar well.

^c^*nd* not determined.

All the studied **AASs** also demonstrated strong antifungal activities against dermatophyte yeasts and fungi. *Trichophyton mantigrophytes* was observed to be the most sensitive dermatophyte followed by *Trichophyton rubrum*, *Candida albicans*, *Trichosporon cataneum*, and *Cryptococcus neoformans*. **HS** displayed the highest antifungal activity followed by **TS,** and **AS**. The **HS** surfactant, having an MLC of 16 µg/ml, resulted in the inhibition zones of 62, 57, 56, 48, and 36 mm diameter against *Trichophyton mantigrophytes*, *Trichophyton rubrum*, *Candida albicans*, *Trichosporon cataneum*, and *Cryptococcus neoformans*, respectively. The **AS** surfactant, having an MLC of 64 µg/ml presented the lowest antifungal potential with inhibition zones of 45, 43, 40, 36, and 35 mm diameter against *Candida albicans*,* Trichophyton rubrum*, *Trichosporon cataneum*, *Cryptococcus neoformans*, and* Trichophyton mantigrophytes*, respectively. Interestingly, the **AASs** antimicrobial activities did not alter when incubated for 10 additional days and had the same diameter of clear zones. Furthermore, the culturing of fresh broth media and agar plates with a loop from these clear zones was unable to grow. These findings confirm the lethality of **AASs** rather than the inhibitor effect. Broad-spectrum antimicrobial activities of **AASs** suggest their non-specific mechanisms as compared to traditional antibiotics, which exhibit specific activities such as cell membrane alteration, antimetabolite activity, and inhibition of protein, cell wall, and nucleic acid synthesis. The **AASs** have been reported to preferably associate with cell membranes rather than targeting a specific process and molecule, which facilitates them to enter the hydrophobic lipid bilayer of pathogenic cells to cause lysis, depolarization, and death. This phenomenon helps to avoid bacterial resistance^25,34,35,67–69^. The interaction of cationic **AASs** with microorganisms mainly occurs through two steps. Initially, amphiphile attaches to the membrane through electrostatic interaction of positively charged surfactant polar head and negatively charged bacterial membrane molecules such as lipoteichoic acid and lipopolysaccharides in Gram-positive and Gram-negative bacteria, respectively. Then, the hydrophobic alkyl chain of **AASs** cationic amphiphiles interacts with membrane lipid bilayers to disrupt its structure and promote the transportation of intracellular materials through the cellular membrane. During the next step, surfactant polarity and hydrophobicity establish an optimum link that helps surfactant diffusion in the lipid bilayer’s non-polar environment^70–72^. This is how cationic surfactants exert their antimicrobial activities. Surfactants are generally more efficient against negatively charged lipids containing Gram-positive bacteria. Surfactants mostly do not exhibit antifungal properties as their negatively charged density in the cell membrane is lower than bacteria^70^. **HS** exhibited significantly high broad-spectrum antibacterial potential against foodborne pathogenic bacteria (*Shigella dysenteriae, Bacillus cereus*, *E. coli*, *K. Pneumoniae,* and *S. aureus*) and fungi (*Trichophyton mantigrophytes*, *Trichophyton rubrum, Candida albicans, Trichosporon cataneum,* and *Cryptococcus neoformans*). **HS** had a low MLC range of 32 and 128 μg/ml. Pinazo et al.^34^ have also reported an optimal link between hydrophobic moiety and **AASs** cationic charge that explains its antimicrobial (yeast, fungi, and bacteria) activities. The **HS** surfactant comparatively exhibited better broad-spectrum antimicrobial activity against dermatophytes that could be compared with antibiotic standards used in this study (Tables 5 and 6). Despite the highly efficient antimicrobial potential, certain drawbacks such as higher production costs are also associated with **AASs** chemical synthesis, which hinders their large-scale applications.

## Conclusions

The investigated **AASs** proved to be efficient copper corrosion inhibitors in 1.0 M H_2_SO_4_ solution at 298 K. The inhibition efficiencies (% IEs) varied with surfactant concentrations and structures and the concentration of the corrosive medium. Higher % IEs of the examined surfactants were attributed to the potential adsorption of surfactant molecules on the copper surface in agreement with the Langmuir adsorption isotherm. The kinetics and mechanisms of corrosion of copper and its inhibition by **AASs** were investigated and discussed. The studied surfactants behaved like mixed-kind inhibitors with slight anodic priority. The results of all the adopted techniques were aligned with each others confirming the validity of the results. **AASs** exhibited good surface activity, emulsifying properties, and significant broad-spectrum antimicrobial activity against pathogenic bacteria and dermatophyte fungi. These features make them ideal candidates for food, pharmaceutical, and personal care product applications.

## Data Availability

All data generated or analyzed during this study are included in this published article.
